# Does Walkability Contribute to Geographic Variation in Psychosocial Distress? A Spatial Analysis of 91,142 Members of the 45 and Up Study in Sydney, Australia

**DOI:** 10.3390/ijerph15020275

**Published:** 2018-02-06

**Authors:** Darren J. Mayne, Geoffrey G. Morgan, Bin B. Jalaludin, Adrian E. Bauman

**Affiliations:** 1 Sydney School of Public Health, The University of Sydney, Sydney, NSW 2006, Australia; geoffrey.morgan@sydney.edu.au (G.G.M.); adrian.bauman@sydney.edu.au (A.E.B.); 2 Public Health Unit, Illawarra Shoalhaven Local Health District, Wollongong, NSW 2502, Australia; 3 School of Medicine, University of Wollongong, Wollongong, NSW 2522, Australia; 4 Illawarra Health and Medical Research Institute, University of Wollongong, Wollongong, NSW 2522, Australia; 5 University Centre for Rural Health—North Coast, The University of Sydney, Sydney, NSW 2006, Australia; 6 Ingham Institute, University of New South Wales, Sydney, NSW 2052, Australia; b.jalaludin@unsw.edu.au; 7 Epidemiology, Healthy People and Places Unit, Population Health, South Western Sydney Local Health District, Liverpool, NSW 1871, Australia

**Keywords:** disease mapping, geographic variation, psychosocial distress, spatial analysis, walkability

## Abstract

Walkability describes the capacity of the built environment to promote walking, and has been proposed as a potential focus for community-level mental health planning. We evaluated this possibility by examining the contribution of area-level walkability to variation in psychosocial distress in a population cohort at spatial scales comparable to those used for regional planning in Sydney, Australia. Data on psychosocial distress were analysed for 91,142 respondents to the 45 and Up Study baseline survey between January 2006 and April 2009. We fit conditional auto regression models at the postal area level to obtain smoothed “disease maps” for psychosocial distress, and assess its association with area-level walkability after adjusting for individual- and area-level factors. Prevalence of psychosocial distress was 7.8%; similar for low (7.9%), low-medium (7.9%), medium-high (8.0%), and high (7.4%) walkability areas; and decreased with reducing postal area socioeconomic disadvantage: 12.2% (most), 9.3%, 7.5%, 5.9%, and 4.7% (least). Unadjusted disease maps indicated strong geographic clustering of psychosocial distress with 99.0% of excess prevalence due to unobserved and spatially structured factors, which was reduced to 55.3% in fully adjusted maps. Spatial and unstructured variance decreased by 97.3% and 39.8% after adjusting for individual-level factors, and another 2.3% and 4.2% with the inclusions of area-level factors. Excess prevalence of psychosocial distress in postal areas was attenuated in adjusted models but remained spatially structured. Postal area prevalence of high psychosocial distress is geographically clustered in Sydney, but is unrelated to postal area walkability. Area-level socioeconomic disadvantage makes a small contribution to this spatial structure; however, community-level mental health planning will likely deliver greatest benefits by focusing on individual-level contributors to disease burden and inequality associated with psychosocial distress.

## 1. Introduction

Mental illness is a leading cause of disability worldwide [1] accounting for 19% of total years lived with disability (YLD) and 7% of disability-adjusted life years (DALY) [2,3] of which 53% is due to depressive and anxiety disorders [4]. Just under one-half (45.5%) of the Australian adult population report having ever experienced a mental disorder in their lifetime and one-fifth (20%) in the previous 12 months [5]. In 2012, the World Health Organization challenged its member states to reduce their disability burdens due to mental illness through coordinated action between health and social sectors [6]. This was followed in 2013 by a comprehensive action plan that emphasised addressing the many determinants of mental illness, including environmental factors that contribute to individual and population-level vulnerabilities [7].

Walkability describes the capacity of the built environment to facilitate walking for various purposes, including transportation, health and leisure [8]. A small but growing literature has emerged over the last decade examining associations between walkability and mental health [9,10,11,12], leading some commentators to recommend walkability as a potential focus for community-level mental health planning and programming [13]. The current evidence base is insufficiently developed to identify a pathway by which walkability may influence mental health; however, two possibilities have been suggested. The first hypothesises that walkable environments help to promote positive affect by increasing participation in moderate-intensity physical activity, such as walking [9]. This is consistent with review findings that participation in regular physical activity protects against the onset of depression and anxiety in healthy populations, and reduces the severity of symptoms in clinical populations [14,15,16]; possibly by modulating melatonin production, adenosine metabolism, and circadian rhythms, or activating brain centres that help reduce negative affect [14]. The second hypothesises that walkable environments may enhance the social capital of neighbourhoods by providing unstructured opportunities for social interactions between individuals [17] that promote trust, and enhance feelings of familiarity, certainty, resilience, and reciprocity [9,17,18,19]. Social capital is understood to buffer individuals against depression and anxiety by reducing daily pressures and promoting health-enhancing behaviours [20]. However, despite their plausibility, neither hypothesis is currently supported by evidence from an appropriate causal evaluation.

Walkability is typically derived as an objective index within a geographical information system [21] using spatial data on residential dwelling density, street network connectivity, land use mix, and—when available—retail destinations, density or floor space [8,22,23]. Indexes originating out of the North American Neighborhood Quality of Life Study (NQLS) [22] and Australian Physical Activity in Localities and Community Environments (PLACE) Study [8] projects have contributed to an extensive evidence base within the transportation, planning, and public health literatures linking the walkability of built environments to improvements in health behaviours and outcomes [24,25,26,27,28,29]. Much of this evidence comes from individual-level studies of participants and the micro (personal) and meso (neighbourhood) environments in which they live [21,30]. However, there is increasing interest in meso (area) environment walkability, its contributions to the distribution of health within populations, and how it may be used to inform population health programming at larger regional scales [8,23,30,31,32].

Psychosocial or psychological distress describes anxious or depressed mood in the absence of a specific psychiatric diagnosis [33] and is commonly used to monitor mental health status in populations using representative surveys [34], such as the United States (US) Behavioral Risk Factor Surveillance System [35] and Australian Health Survey [36]. Environmental influences on mental health have received considerable attention in the research literature (see [19] for reviews); however, only a small number of studies have directly addressed relations between walkability and mental health outcomes [19], and none at the spatial scales typically used for population health planning and intervention. Between-group analyses of outcomes such as psychosocial distress can identify population sub-groups at increased risk of adverse mental health outcomes but provide limited information on the geography of these risks. In contrast, spatial analyses may be used to identify areas at increased risk of adverse outcomes or spatially structured influences on health by focusing on geographic variation in excess of that due to known demographic, social, economic, and health factors [30,37,38].

Spatial analyses of health outcomes and behaviours are increasingly common in the epidemiological literature as statistical methods and geographically-referenced administrative, surveillance and research data become more accessible [39]. Spatial analyses are especially informative for population health programming [30], which typically occurs at larger, regional spatial scales [31]. For example, Chaix et al. identified differing spatial distributions and cluster resolutions of psychoactive substance use and neurotic disorders in Malmö, Sweden, which were associated with adverse social environments [40]. In addition to identifying potential contextual factors for public health action, the analysis also established appropriate levels for intervention by characterising the spatial scales at which variations in mental health outcomes occur [40]. Likewise, Cheung et al. [41] and Ngamini Ngui et al. [42] have reported spatial heterogeneity in suicide across Australia and Québec, Canada, and conclude that understanding this variation is essential to framing national and regional mental health policy. Spatial analysis has also been instrumental in describing geographic variation in psychological susceptibility and its association with resilience factors after Hurricane Sandy in New York City [43].

The objective of this study was to assess the contribution of walkability to geographic variation in mental health outcomes at spatial scales typically used for population-level health programming, planning, and intervention. It builds on our previous work demonstrating the contribution of area-level walkability to geographic variation in population-levels of total walking and moderate and vigorous-intensity physical activity [30]. Our aims were to: (1) evaluate if area-level walkability was associated with area-level psychosocial distress; (2) describe geographic variation in area-level psychosocial distress; (3) assess the contribution of individual-level factors to geographic variation in area-level psychosocial distress; and (4) quantify the contribution of area-level walkability to geographic variation in area-level psychosocial distress not attributable to person-level characteristics using a population-based cohort living in Sydney, Australia. We hypothesised that (1) areal-level psychosocial distress would be spatially structured, and that (2) at least some of this structure would be attributable to area-level walkability.

## 2. Materials and Methods

### 2.1. Study Design and Area

We used a cross-sectional, ecological design to investigate geographic variation in psychosocial distress and its relationship to walkability in the Sydney Statistical Division of New South Wales, Australia [44]. Sydney covers a land area of 12,142 km^2^ and had a population of 4.1 million persons living in 1.6 million dwellings at the 2006 Australian Census [45]. Analysis was undertaken at the Australian Census of Population and Housing postal area level to coincide with the finest spatial resolution at which the data custodian provided geographical identifiers for 45 and Up Study cohort members. There were were 260 postal areas in Sydney in 2006 [46] with a median land area of 7.6 km^2^, 5304 residential dwellings and 13,090 residents [45]. This land area is equivalent to a radial buffer of 1550 m, and corresponds with the upper level of high-resolution buffers used in individual-level studies for which consistent environment-behaviour associations have been reported [47,48].

### 2.2. Participants

Participants for this study were drawn from The Sax Institute’s 45 and Up Study [49]. The 45 and Up Study is a population-based cohort established to investigate health ageing among persons aged 45 years and over in New South Wales, Australia [49]. Recruitment into the study began in January 2006 and was finalised in December 2009 [50] with a total cohort size of 267,153 or 10% of the New South Wales population aged 45 and over [51]. Potential participants were randomly sampled from the Department of Human Services (formerly Medicare Australia) enrolment database, and included an oversample of persons aged 80 years and over. People living in rural areas were also oversampled, and all residents from remote areas were invited to participate [49]; however, neither of these population subgroups are represented in the Sydney Statistical Division. Selected individuals were mailed an invitation letter, and asked to return a signed, written consent form with their baseline survey via reply-paid mail if they consented to participating in the study [49]. We were provided access to the April 2010 data release comprising 266,848 participants [52], which the data custodian had geocoded to 2006 Australian Standard Geographic Classification Statistical Divisions [44] and postal areas [46]. We limited our analysis to participants geocoded to the Sydney statistical division of New South Wales to coincide with the spatial extent of our study factor.

### 2.3. Data

Individual-level data comprised self-reported responses to the baseline questionnaire of the 45 and Up Study [49], and were used to derive respondent-level outcomes and covariates. Postal area data included the Sydney Walkability Index [23] and 2006 Index of Relative Socioeconomic Disadvantage [53], which were included as study and covariate factors, respectively.

### 2.4. Outcome Variable

Psychosocial distress served as the outcome factor in our analysis, and was measured using the Kessler Psychological Distress Scale (Kessler 10) [54]. The Kessler 10 is a dimensional measure of non-specific psychosocial distress developed to discriminate between cases and non-cases of serious mental illness in community populations [54]. The scale comprises 10 questions that ask respondents to rate how frequently over the past four weeks they felt tired for no good reason; nervous; so nervous that nothing could calm them down; hopeless; restless or fidgety; so restless that they could not sit still; depressed; that everything was an effort; so sad that nothing could cheer them up; and worthless [54]. Item responses are scored from 1 (none of the time) to 5 (all of the time) and then summed to give a total between 10 and 50. In Australia, scores of 22–29 and ≥30 are sensitive and specific for high and very high levels psychosocial distress in community populations, respectively [55]; specific for any current anxiety or affective disorder [56]; and associated with other mental disorder categories, and presence of any current mental disorder [56]. We created a single, binary outcome variable and classified individuals with a total scale score ≥22 as having high (or very high) psychosocial distress for consistency with existing state and national representative surveys monitoring population levels of psychosocial distress [55,57,58,59]. List-wise exclusions due to incomplete item responses were minimised by imputing invalid and missing data using the pairing up and mean substitution methods implemented in the Household, Income and Labour Dynamics in Australia Survey [60].

### 2.5. Study Variable

The primary variable of interest for all analyses was postal area walkability, which we measured using the Sydney Walkability Index. [23]. This index is a three-factor index derived using methods and data comparable to the Neighborhood Quality of Life Study (NQLS) and Physical Activity in Localities and Community Environments (PLACE) walkability indexes [8,22], both of which underpin extensive national and international literatures [23]. The Sydney Walkability Index is calculated within a geographical information system using three built environment variables:

1. Residential dwelling density—the number of residential dwellings per square kilometre of residential land use
2. Intersection density—the number of intersections with three or more roads per square kilometre of total land area
3. Land use mix—the entropy of residential, commercial, industrial, recreational and other land uses.

Environmental variable values are divided into deciles, scored from 1 (lowest) to 10 (highest), summed to give a total score out of 30, and then divided into quartiles corresponding to low, low-medium, medium-high and high walkability [23]. We have previously demonstrated the predictive validity of the Sydney Walkability Index for utilitarian walking, and its comparability to four-variable indexes (e.g., [8,22]) found in the research literature [23]. We have also recently reported positive associations between the Sydney Walkability Index and population-levels of sufficient walking and total moderate and vigorous-intensity physical activity to enhance health, and its contribution to geographic variation in physical activity behaviours between postal areas in the Sydney statistical division [30].

### 2.6. Covariates

We included a number of individual- and area-level correlates of psychosocial distress previously identified for the 45 and Up Study cohort in the research literature [61,62,63,64,65,66,67,68,69,70,71,72,73,74]. Individual-level covariates included sex (male, female); five-year age group at baseline interview (45–49 to 80–84 and ≥85 years); language spoken at home (English, other); educational level (less than secondary school, secondary school graduation, trade or certificate or diploma, university degree); relationship status (partner, no partner); employment status (full-time, part-time, other, not working); health insurance type (private with extras, private without extras, Government health care card, none); smoking status (never, past, current), World Health Organisation body mass category (underweight <18.5 kg/m^2^, normal weight 18.5 to <25.0 kg/m^2^, overweight 25.0 to <30.0 kg/m^2^, obese ≥30.0 kg/m^2^); moderate and vigorous-intensity physical activity in the previous seven days (0, 1–149, 150–299, ≥300 min); number of chronic conditions ever diagnosed and treated in the previous four weeks (0, 1, 2, 3 or more); and limitations on physical functioning (none, minor, moderate, severe). All data were obtained by self-report. Limitations on physical functioning were measured using the Medical Outcomes Study (MOS) 36-Item Short-Form Health Survey (SF-36) physical functioning scale [75,76], with scores of 0 to <60, 60 to <90, 90 to <100, and 100 classified as none, minor, moderate, and severe respectively. Socioeconomic disadvantage was measured at the postal area level using the 2006 Index of Relative Socioeconomic Disadvantage [53]. This index is a general measure of disadvantage derived by principal components analysis of 2006 Australian Census of Population and Housing Census variables indicative of low socioeconomic status (see [53]).

### 2.7. Statistical Analysis

Our analysis utilised a two-step approach to model relative prevalence within the study cohort. In the first step, the predicted probabilities (${\hat{Y}}_{ij}$) of psychosocial distress were estimated for each person from fixed-effect logistic regression models conditioned on individual-level social, economic and health factors as model covariates. We then summed the predicted probabilities for the *j* postal areas to obtain the total expected numbers of persons with psychosocial distress in each postal area adjusted for its underlying respondent structure (see [30,77,78,79]).

In the second step, we used used Bayesian Besag, York and Mollié conditional auto regressive models with Poisson likelihoods to estimate prevalence ratios for each of the *j* postal areas relative to the study area [80]. Besag, York and Mollié spatial models decompose area-level random effects into local, spatially structured ($s_{j}$) and global, unstructured ($u_{j}$) variance components [81,82] using:

(1) $$log\left(\theta_{j}\right)=\alpha +x_{j}\beta +s_{j}+u_{j}+log\left(e_{j}\right)$$

where $\theta_{j}$ is the prevalence ratio for the $j^{th}$ postal area; α is the mean prevalence ratio for the study area; $x_{j}$ and β are optional vectors of ecological explanatory variables and parameter estimates, respectively, and $e_{j}$ is a model offset representing the expected number of cases in the $j^{th}$ area. The unstructured variance component was given a normal prior with mean 0 and precision $\tau_{u}^{2}$, while the spatial variance component used an intrinsic conditional auto regressive prior [81] with mean ${\bar{s}}_{j}$ and precision $tau_{j}^{2}$ conditioned on the prevalence in the surrounding *k* postal areas with contiguous boundaries [81]. The hyper-parameters $\tau_{u}^{2}$ and $\tau_{s}^{2}$ were used to control the variability of $u_{j}$ and $s_{j}$, and were given Gamma hyper-priors of γ(0.5,0.0005) [83]. We derived expected cases $e_{j}$ using either the overall prevalence ($e_{j}=p\times n_{j}$) for unadjusted models or the sum of the predicted probabilities from stage one ($e_{j}=\sum {\hat{Y}}_{ij}$) in the case of models adjusted for individual-level factors (see [30,77,78,79]).

Our analysis fit six analytic and two sensitivity models. Model 1 (M1) was an unadjusted disease mapping model with offsets proportional to the study area prevalence ($p\times n_{j}$). Model 2 was also a disease model but with individually-adjusted offset terms from stage 1 models. Models 3–6 were ecological regressions: model 3 added postal area walkability to model 2; model 4 added postal area socioeconomic disadvantage to model 2; and model 5 included individually-adjusted offsets, postal area walkability, and postal area socioeconomic disadvantage. Model 6 tested for effect modification of the relationship between psychosocial distress and walkability by socioeconomic disadvantage. We additionally assessed the sensitivity of our association between walkability and psychosocial distress to excluding physical activity level from fixed-effects models used to adjust spatial regression offset terms for individual-level characteristics. These analyses acknowledge the uncertainty regarding the path between walkability and psychosocial distress. If this were mediated by physical activity, as implied by the possible route suggested by Sturm et al. [9], then adjusting for physical activity may suppress the substantive association between walkability and psychosocial distress. We assessed this possibility by refitting models 2 and 3 after excluding physical activity level from the fixed-effect model used to adjust spatial regression offset terms.

Medians and 95% credible intervals for each model parameter were summarised from the posterior distributions of two Monte Carlo Markov Chains initialised using over-dispersed starting values. We ran each chain for 2.5 million iterations and retained every 250th sample to reduce autocorrelation and improve convergence. We discarded the first half of each chain as burn-in, giving 10,000 samples in total for inference. Autocorrelation plots and the Gelman-Rubin diagnostic [84] were used to confirm the convergence of MCMC chains [85]. All models were fit using unweighted survey data, which produce representative and generalisable relative effect estimates for individual-level analyses [86] and unbiased relative effect estimates for postal area analyses [30] in this cohort.

We used the *Deviance Information Criterion* (DIC) to choose between competing conditional auto regressive models with smaller values taken as evidence for improved fits [87]. We also exponentiated and mapped the linear predictor, and spatial and non-spatial random effects for postal areas to identify variation in excess of that attributable to individual- and area-level factors. We additionally calculated spatial fractions ($\rho =\sigma_{s}^{2}/[\sigma_{s}^{2}+\sigma_{u}^{2}]$) from the marginal variances of the random effects to estimate the proportion of residual variation in high psychosocial distress due to unobserved and spatially-structured factors (see [88,89]). All data analysis and mapping was undertaken in R 3.3.2. Fixed effects logistic regressions were evaluated at the 5% alpha level and conditional auto regressive Poisson regressions using 95% credible intervals summarised from posterior distributions.

### 2.8. Ethical and Data Access Statements

The 45 and Up Study is approved and monitored by the University of New South Wales Human Research Ethics Committee (ref no. HREC 05035/HREC 10186). The present research comprised a sub-study of the Social, Environmental, and Economic Factors Study, which is approved and monitored by the University of Sydney Human Research Ethics Committee (ref no. 10-2009/12187). Details on accessing 45 and Up Study data are available on the The Sax Institute website (www.saxinstitute.org.au/our-work/45-up-study).

## 3. Results

Complete data were available for 91,142 of 115,153 (79.1%) Sydney respondents residing in 254 of 260 (97.7%) study postal areas. The median number of respondents per postal area was 258, with a minimum of 0, maximum of 3302, and inter-quartile range of 145–441 respondents. Table 1 shows individual characteristics for respondents included in our analysis. Similar to the full 45 and Up Study cohort [52], our sample had similar gender and employment characteristics to the study area but was otherwise younger, more highly educated, less likely to speak a language other than English at home, and more likely to be living with a partner than the Sydney population aged 45 years and over [45].

### 3.1. Walkability

We have previously reported in detail on built environment variables and walkability profiles for Sydney postal areas [30]. Environmental variables increased monotonically for low, low-medium, medium-high and high walkability postal areas: residential density (2.3, 13.4, 19.8 and 46 dwellings per hectare), street network connectivity (3.4, 46.1, 79.5 and 162.5 intersections per square kilometre), and land use mix entropy (0.005, 1.033, 0.056, and 0.134), and walkability was distributed along an east-west gradient with highest concentrations of walkable areas surrounding and north of the Sydney central business district, and lowest concentrations in Western Sydney and the peri-urban fringe [23,30].

### 3.2. Prevalence of Psychosocial Distress

The within cohort prevalence of high psychosocial distress was 7.8% (7.6–8.0%). Prevalence estimates by postal area characteristics are reported at the top of Table 1. Levels of high psychosocial distress were similar in low, low-medium, and medium-high walkability areas, and slightly lower in high walkability areas. In contrast, prevalence of high psychosocial distress decreased monotonically with decreasing relative socioeconomic disadvantage, and was 2.6 times lower in least versus most disadvantaged areas.

### 3.3. Spatial Analysis

**Figure 1:**
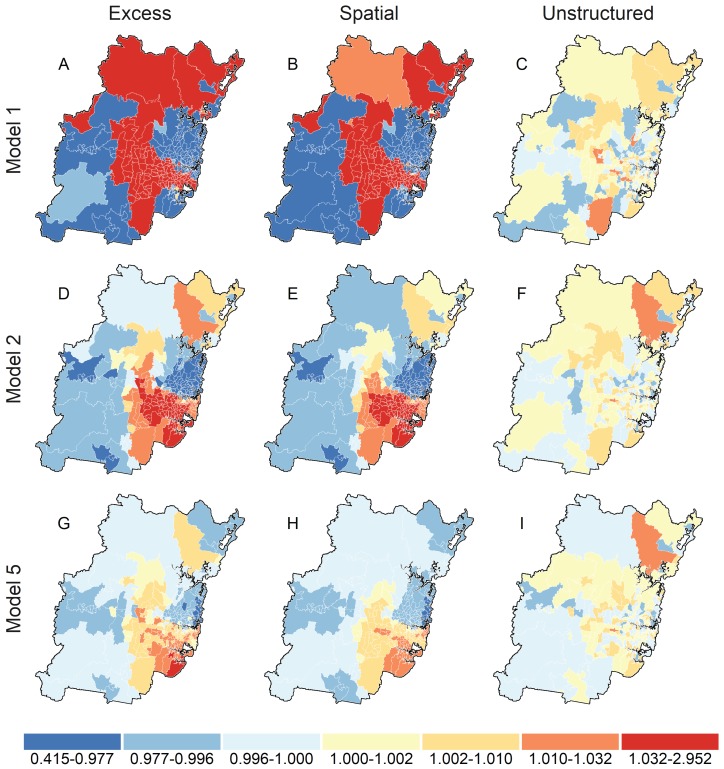
Total, Spatial and Unstructured prevalence ratios for Sydney postal areas. Total prevalence ratios were derived by exponentiating the sum of the log odds for the *s* and *u* random effects; Spatial and Unstructured prevalence ratios were obtained by exponentiating the log odds of the individual *s* and *u* components, respectively. Total, Spatial, and Unstructured prevalence ratio estimates are reported in maps **A**–**C** for model 1, maps **D**–**F** for model 2, and maps **G**–**I** for model 5.

Map A in Figure 1 reports the smoothed distribution of unadjusted prevalence ratio for high psychosocial distress in Sydney statistical division estimated from model 1. There is strong evidence for clustering of high psychosocial distress with a band of relatively higher prevalence postal areas stretching from the north, through the centre, and then to the south-eastern border of Sydney. Prevalence ratios were consistently lower for postal areas in the central business and surrounding districts on the eastern seaboard, and in south western Sydney. Maps B and C decompose the total prevalence into its spatial and unstructured sources, respectively. Map C indicates that little variation is due to unstructured factors, while map B shows that the distribution of high psychosocial distress is largely attributable to unobserved and spatially-structured factors. This is confirmed by the Model 1 spatial fraction reported in Table 2, which attributes almost all of the variation in map A to the spatial random effect.

Table 3 reports unadjusted odds ratios (OR) for associations between high psychosocial distress and individual-level covariates, which were used to adjust expected values in spatial models. All variables were statistically significant and important in univariate models with small to medium effect sizes [90]. Odds for high psychosocial distress were increased for females, people who spoke a language other than English at home, had less than a university education, were not working full-time, did not have private health insurance, or were on a government health care card. Higher odds were also observed for current and past smokers, persons who were underweight or obese, had one or more chronic conditions ever diagnosed or treated in the last month, or experienced minor to severe physical limitation. Reduced odds of high psychosocial distress were associated with older age, peaking in ages 65–74, and longer durations of total moderate and vigorous-intensity physical activity per week.

Adjusted OR remained important but were attenuated relative to unadjusted effect estimates (see Table 3). The two exceptions were age and body mass category. The protective effect of age relative to persons 45–49 year became stronger throughout the life span following adjustment, peaking in the 80–84 years age group, while odds of high psychosocial distress for obese relative to normal weight individuals switched from 1.64 (1.54–1.75) to 0.88 (0.82–0.94). The latter was due to confounding of the association by limitations on physical functioning, age, and number of chronic conditions ever diagnosed. Obese respondents with high psychosocial distress were more likely to have severe functional limitations (50.2% versus 35.1%) or been diagnosed with three or more chronic health conditions (24.8% versus 13.5%), and less likely to be aged 80 years or older (4.9% versus 11.5%) compared to non-obese persons.

The second row of maps in Figure 1 shows relative prevalence of high psychosocial distress (map D), decomposed into to spatially structured (map E) and unstructured (map F) factors after accounting for individual-level differences between Sydney postal areas (model 2). The magnitude of prevalence ratios were substantially attenuated and reduced in range from 0.42–2.92 for model 1 to 0.86–1.09 for model 2. Despite this reduction, prevalence ratio remained geographically clustered with higher rates in central and south-eastern Sydney, and lower rates in north Sydney (see maps D–F). The DIC and pD for model 2 indicated a substantially better fit over model 1, which reduced spatial and unstructured variation by 98.5% and 52.1%, respectively, and the spatial fraction by 11.1% (see Model 2 in Table 2).

Associations between high psychosocial distress and postal area walkability (model 3) and relative socioeconomic disadvantage (model 4) are reported in Table 2. We found no evidence for an association between psychosocial distress and postal area walkability after adjusting for individual-level factors. The DIC and pD for model 3 indicated a poorer fit compared to model 2, and all walkability credible intervals included unity. Excluding physical activity level from model offsets in sensitivity analyses did not alter prevalence ratios obtained from model 3 (see Table 4). The increase in DIC (0.98) and pD (2.39) for this sensitivity model relative to a baseline sensitivity model excluding walkability and physical activity also provided no support for an association between psychosocial distress and walkability, or excluding physical activity from our analysis (see Table 4). Model 4 added relative socioeconomic disadvantage to model 2, which also included individual-level socioeconomic factors, resulted in an improved model that reduced DIC by 9.3 units and pD by 6.4 parameters. Compared to postal areas in the most socioeconomically disadvantaged quintile 1, prevalence of high psychosocial distress was similar for postal areas in quintile 2, and 8%, 10% and 18% lower for postal areas in quintiles 3–5, respectively (see Table 2).

The bottom row of Figure 1 displays final prevalence ratios from model 5 for high psychosocial distress (map G) decomposed into spatially structured (map H) and unstructured (map I) factors after accounting for individual differences, and postal area walkability and socioeconomic disadvantage. Simultaneously adjusting for individual and postal area factors further attenuated prevalence ratios but did not substantially affect the geographic distribution of high psychosocial distress, which remained higher-than-expected in central and south-eastern Sydney, and lower-than-expected in north Sydney. Adjusting for relative socioeconomic disadvantage in model 5 did not alter effect estimates or conclusions for the association between postal area walkability and high psychosocial distress from model 3 (see Table 2). The DIC value for model 5 was 1.3 units larger than the “best” fitting model 4 but within the ≤2 unit change range indicating a model deserving consideration [87]. Spatial and unstructured variation in fully adjusted model 5 were reduced by 99.9% and 59.1% relative to unadjusted model 1, and the spatial fraction reduced from 0.99 to 0.55 (38.4%). Interaction model 6 provided no evidence that the association between walkability and high psychosocial distress was modified by postal area socioeconomic disadvantage ($DIC_{M6}-DIC_{M5}=$ 18.1).

## 4. Discussion

This appears to be the first study to assess associations between area-level walkability and psychosocial distress using a large population cohort within a spatial framework. Our findings indicate that while psychosocial distress is geographically clustered in the Sydney statistical division, area-level walkability does not contribute to this spatial structure, which is principally patterned by the individual-level characteristics of residents within postal areas. We did, however, observe a consistent association between postal area socioeconomic disadvantage and prevalence of high psychosocial distress independent of individual-level social and economic factors. Prevalence of high psychosocial distress is 10–18% lower in the least compared to most socioeconomically disadvantaged postal areas after adjusting for individual-level differences and postal area walkability. Our results suggest that while area-level socioeconomic disadvantage makes a small contribution to geographic variation in psychosocial distress (2.2%), programming and planning activities will likely deliver greatest benefits by focusing on individual-level determinants, correlates, and mediators of disease burden and inequality associated with psychosocial distress.

Modifying the walkability of built environments to improve the health of populations is frequently recommended [8,23,91,92,93,94,95], and has been suggested as a potential focus for community-level mental health planning [13]. Such recommendations implicitly assume that individual-level environment-behaviour and environment-outcome findings scale to community- and population levels. However, these assumptions are rarely evaluated, which leaves open the potential for spurious cross-level action due to atomistic [96] or individualistic [97] fallacy. Our study is novel in that we have directly examined associations between area-level walkability and high psychosocial distress in Sydney at spatial scales more typical of population-level programming, planning, and intervention. At these scales, we observed substantial geographic variation in unadjusted disease maps of psychosocial distress prevalence for postal areas. However, we found no evidence supporting a link between walkability and prevalence of psychosocial distress or its geographic patterning, both of which appear largely attributable to the spatial distribution of individual-level factors across the Sydney statistical division with a small contribution from postal area socioeconomic disadvantage.

An evidence base linking walkability to mental health outcomes is only beginning to emerge in the research literature, and is presently derived from a small number of individual-level studies. Berke et al. reported in 2007 that the odds of depression in the Adult Changes in Thought (ACT) Study cohort were reduced by a factor of 0.31–0.33 for the highest versus lowest walkability quartile but only for older men. In contrast, a 2011 cross-sectional study of older men in Perth, Australia, found that while depression was unrelated to Census Collection District walkability, it was associated with individual environmental variables used to construct their index, with increased odds of depression in Districts with middling (1.10–2.16) and high (1.08–2.14) versus low land use mix, and some versus no retail land use (1.04–1.90) [10]. However, a subsequent study of older Welsh men has reported reduced odds of psychosocial distress for greater land use mix (0.22–1.00) and street network connectivity (0.28–1.00) [11], another built environment variable routinely included in walkability indexes [8,22]. While most recently, James et al have reported that the odds of depression (1.08–1.16) and current anti-depressant use (1.08–1.25) were significantly increased among persons living in the highest versus least walkable neighbourhoods of low-income and racially diverse populations in south eastern United States [12].

The heterogeneity of findings from these studies likely reflects the considerable variability in methods and measures they employed [19]. Walkability was assessed using both objective and perceived methods, and no two studies used the same index, scale or combination of environmental variables to measure walkability. Likewise, mental health outcomes were assessed for a diverse range of conditions and symptoms using a mix of standardised scales and self-report. In their 2016 systematic review, Gong et al. identified an urgent need to develop standardised approaches to researching built environment influences on mental health [19]. This concern reflects a broader focus in the walkability literature to reconcile environment-behaviour research methods to improve between-study comparability and inform public health policy and planning (e.g., [21,23,98]). Gong et al. have also emphasised the importance of utilising objective built environment indexes in mental health research to reduce information bias resulting from a tendency among persons with poorer mental health to perceive their environments more negatively [19]. Our study design is consistent with these recommendations in its use of validated outcome and objective exposure variables, which are routinely used for population-level health surveillance [34], and individual- and area-level walkability research (e.g., [8,22,23,95,99]).

The mechanism by which walkability my influence psychosocial distress remains an important but unresolved issue for current and future environmental exposure research. Both physical activity [9] and social capital [17] have been hypothesised as plausible variables through which environmental walkability acts on mental health; however, neither has been evaluated within a causal framework. Our study indirectly considered the influence of individual-level physical activity on area-level associations between walkability and psychosocial distress through a sensitivity analysis that excluded physical activity from the model used to derive offset terms for ecological regressions. We obtained the same effect estimates for environmental walkability regardless of whether we adjusted for physical activity or not; however, our study design (cross-sectional) and analytic approach (ecological) preclude us from making inferences about the possible mediating role of this variable. Ideally, any evaluation of potentially mediating variables should use prospectively collected data from multiple waves of follow-up to allow sufficient time to elapse between the hypothesised cause and its effect, and to avoid the bias that arises when cross-sectional data are used to estimate longitudinal effects [100]. The 45 and Up Study comprises 265,000 persons aged 45 years and older [49], with 40% residing in a geographical unit classified by the Sydney Walkability Index. Follow-up of this cohort occurs approximately quinquennially, with a third wave of data collection scheduled to begin in the next few years. This will provide a unique opportunity to evaluate potential causal pathways between walkability and mental health, and how they may contribute to healthy ageing.

We observed strong associations between all individual-level socioeconomic indicators and psychosocial distress. This is consistent with the substantive (e.g., [101,102,103,104,105]) and 45 and Up Study literatures (e.g., [68]) indicating higher prevalence of poorer mental health in more socioeconomically disadvantaged individuals, regardless of how mental health and socioeconomic status are measured. Odds of psychosocial distress were 1.05–1.89, 1.27–1.92, and 1.02–1.87 times higher for persons not in full-time work, without private health insurance, and without a university degree, respectively. We also observed a consistent contextual effect of relative socioeconomic disadvantage on prevalence of psychosocial distress that reduced postal area ratios by 10–24% in the least compared to most disadvantaged quintiles. This gradient is supported by a recent narrative review, which reported consistent evidence for a contextual socioeconomic effect over-and-above that due to individual-level socioeconomic factors [106]. In our study, this contextual effect accounts for approximately 2.3% of the spatial and 4.2% of the non-spatial variation in prevalence of postal area psychosocial distress that remains after adjusting for individual-level factors and area-level walkability. This is smaller than the 13.5% of residual unstructured variation in depression prevalence from World Health Organisation health surveys due to country-level income and income inequality reported by Rai et al. [107]; similar to the 4.5% of unstructured variation in depressive symptoms due to area-level mean income and Gini Coefficient reported by Lee at al. for 253 Korean communities [108]; and consistent with review evidence indicating individual-level factors account for most of the unstructured variation between higher-order cluster units [106]. Cross-level interactions between area- and individual-level socioeconomic status were beyond the scope of this study; however, the available evidence suggests that poorer individual-level socioeconomic position increases susceptibility to neighbourhood-level socioeconomic disadvantage, while improved individual-level position buffers against this effect [106].

We also observed very strong associations between psychosocial distress and numbers of chronic conditions ever diagnosed, and psychosocial distress and limitations on physical functioning. The odds of psychosocial distress were 1.6, 2.5, and 4.3 times higher for person with 1, 2 or 3 or more doctor diagnosed chronic conditions than those with none. Similarly, the odds of psychosocial distress among respondents with minor, moderate or severe limitations on physical functioning were 1.2, 2.2, and 4.4 times higher than those with none. These findings agree with previously published studies on the correlates of psychosocial distress and depression among 45 and Up Study participants [64,65], and Australian [109] and international [110] primary care cohorts. Ormel et al. have identified three components to associations between depressive symptoms and functional disability: an immediate effect of decreased physical function on depressive symptoms; a weaker, lagged effect of functional disability leading depressive symptoms; and a weak, lagged effect of depressive symptoms leading functional disability, all of which may be modified by personal resilience factors and access to effective care [111]. The symptoms of depression and psychosocial distress might also be exacerbated by the social stresses and stigma associated with reduced physical function [65]. These possibilities are consistent with reports that psychosocial distress is more strongly related to level of disability among 45 and Up Study participants with cancer than the fact of a cancer diagnosis [62]. Our study employed a cross-sectional design, which precluded us from identifying the directionality of associations between psychosocial distress, multiple chronic diseases, and limitations on physical functioning. However, our findings do support a role for these factors in the geographical patterning of psychosocial distress across the Sydney statistical district, which is likely to be especially informative for planners, policy-makers, and researchers for population-level health programming, intervention, and evaluation activities.

Although we found no evidence for an association between postal area walkability and psychosocial distress, our findings still have relevance for population-level mental health planning. First, our study demonstrates the utility of visualising geographic variation in mental health outcomes to identify areas with higher or lower than expected rates, which may provide targets for population-level intervention. The utility of mapping for service planning has been demonstrated by Bazemore et al., who used geographical information systems to visualise and address discrepancies between services delivered and under-served areas in a North American primary care clinic network [112]. Our findings indicate that psychological distress is geographically clustered in Sydney, and that it is the spatial distribution of individual-level demographic, social, economic and health factors that drive this patterning. From a planning perspective, adding or removing individual-level factors sequentially and visualising their effect on disease maps would be especially informative for identifying those individual-level characteristics and circumstances contributing to higher-than-expected psychosocial distress in a specific geographic area. This was beyond the scope of our research, which was concerned with the contribution of area-level walkability to postal area psychosocial distress prevalence and geographic variation over and above that attributable to individual level factors. We observed no association between postal area psychosocial distress and walkability, and walkability had little effect on disease maps. This suggests area-level walkability is insufficiently sensitive for informing population health policy and programming aimed at improving mental through built environment intervention, and that planners and policy-makers are more likely to maximise health gains by focusing on established individual-level correlates and determinants of mental (ill) health.

A major strength of our study is it use of the large, high-quality 45 and Up Study cohort, which has population-level coverage. However, similar to the larger cohort, our sample was younger, better educated, and more likely to be partnered and speak English at home than the general population aged 45 years and over in the Sydney statistical district. While this precludes us from generalising point-prevalence estimates beyond our sample, it is likely that our relative effect estimates are externally valid. It is well established in the epidemiological literature that relative measures of risk and odds derived from cohorts are usually generalisable irrespective of representativeness and non response [113,114]. This has been specifically demonstrated in the case of the 45 and Up Study by Mealing et al., whom reported that odds ratio estimates from this cohort are highly comparable to those derived from the population-representative New South Wales Continuous Health Survey [86]. We have also reported very high correlations between postal area relative risks and disease maps estimated from unweighted and post-stratification weighted data, which indicates spatial risk estimators within the 45 and Up Study cohort are unaffected by non response bias [30,115].

Our study used validated measures for quantifying outcomes and exposures. The Kessler 10 [54] is an established, scale-derived measure of psychosocial (psychological) distress that is routinely used in research and to monitor mental health status in population-representative surveys [34], making it an ideal choice for our application. Similarly, the Sydney Walkability Index is an established indicator of the built environment with demonstrated validity and specificity for walking behaviour at a range of spatial scales [23,30]. Objectively characterising the walkability of built environments is especially important for mental health outcomes where systematic information bias is reasonably expected [19]. In addition to being objective, the Sydney Walkability Index is constructed using the same methods as other influential indexes in the walkability literature (see [8,22]). However, while our index is derived at the area-level, we caution against interpreting it as a proxy for individual-level exposure [30]. We deliberately matched the spatial scales at which we measured outcome and exposure variables to avoid validity concerns arising from cross-level inference [116], which was evident in at least one of the individual-level studies reviewed (see [10]). We argue that when walkability exposure and outcome are measured at the same area-level resolution, it constitutes a contextual variable describing the shared walkability experience of populations and groups inhabiting the same geographic space [30]; we have demonstrated the plausibility of this conceptualisation using the same cohort and spatial scale (see [30]). We believe this makes our approach especially relevant to planning applications, which typically occur at regional levels and for populations of individuals.

Another strength of our study is its use Bayesian Besag, York and Mollié spatial models fit as disease mapping and ecological regressions to: (1) directly assess associations between outcomes, exposures, and covariates; (2) quantify geographic clustering of high psychosocial distress; and (3) evaluate the contribution of postal area walkability to this spatial structure. Spatial methods are increasingly employed in the epidemiological literature to understand the role of place on health outcomes, behaviours and determinants, and to account for spatial autocorrelation, which is problematic for valid inference if not handled appropriately [117]. Our study demonstrates the highly spatial nature of psychosocial distress in Sydney and the importance of handling this geographic structure at the analysis stage. While standard multilevel analysis can account for autocorrelation through random effect terms, our study highlights the advantage of decomposing this variation into spatial and non-spatial sources for informing programming, planning, and intervention activities. We also avoided potential confounding in our analysis due to individual differences in the underlying response populations by adjusting model offsets using predicted probabilities from individual-level fixed-effects regressions of psychosocial distress on person-level demographic, social, economic and health factors. This approach is commonly employed in the epidemiological literature to adjust area-level models where individual-level variables cannot be parameterized within a parsimonious model [78] or would be computationally prohibitive [30,77,79].

Our study is subject to a number of limitations. We were unable to include a measure of social social capital in our study despite its hypothesised link with walkability and psychosocial distress. Self-reported measures of social capital were collected as part of the 45 and Up Study baseline survey but were poorly completed. Limiting our analysis to cases with complete data on these variables would have further reduced our effective sample size, and resulted in a non-response rate well above the maximum 20% identified for cohort studies and data that are missing not at random (MNAR) (see [118,119]). However, we do not believe including social capital would have substantially altered our findings for two reasons. First, we observed no association between walkability and psychosocial distress for social capital to be considered a potential mediator [120]. And second, a recent individual-level study of the association between walkability and mental health reported that effect estimates were unchanged when social capital was included in statistical models [12], which is inconsistent with a moderating effect by social capital [121].

Another limitation of our study is that individual- and area-level factors were modelled separately. Ideally, all variables would be included in a single, parsimonious model that allowed their joint effects to be assessed concurrently. These types of multi-level spatial models are beginning to emerge in the epidemiological literature (e.g., [122]) but are not easily implemented in standard statistical software, and are often computationally prohibitive for problems with large sample sizes and numerous spatial units outside of high performance computing environments [122]. Our approach to adjusting spatial models using offset terms derived from fixed-effect analyses of individual-level factors is commonly employed in the epidemiological literature where a parsimonious model cannot be specified or is computationally prohibitive [30,77,78,79], as was the case in this study. However, recent methodological advances incorporating Integrated Nested Laplace Approximation (INLA) to estimate approximate posteriori marginals appear to offer a potential solution for the efficient fitting of these multi-level Bayesian spatial models [123,124].

Finally, our study used Australian-specific postal areas as the units of analysis, and sample-specific cut-points for the calculation of Sydney Walkability Index variables. The spatial extents of postal areas may not coincide with the planning units used in other jurisdictions. Associations between outcomes and exposures can vary with geographic resolution, even when both are measured at the same spatial scale [125]. As such, this should be taken into consideration when applying our findings at finer or coarser spatial scales. However, we do note that the median land area of our postal areas was 7.6 km^2^ or the equivalent of a 1550 m radial buffer, which is at the upper limit of buffer sizes used in individual-level studies, and for which consistent environment-behaviour associations have been reported [47,48]. We also quantized environmental variables relative to their distribution in the Sydney statistical division, which may not be representative of other jurisdictional spatial units. To address this potential limitation we have reported the cut-points used to construct our index [30], and encourage planners, policy-makers, and researchers to use these in assessing the applicability of our results to their setting of interest. We also acknowledge that the cross-sectional design of our study limits its conclusions to non-causal inferences.

## 5. Conclusions

Walkability describes the capacity of the built environment to promote or hinder walking for multiple purposes, and has been proposed by Berke and colleagues as a potential environmental focus for mental health planning and intervention [13]. Our study examined this possibility at a spatial scale similar to those typically used for regional-level planning and found no evidence for an association between postal area walkability and high psychosocial distress in the Sydney Statistical Division that could be leveraged for this purpose. We did, however, observe strong geographic clustering of high psychosocial distress, which was largely attributable to individual-level factors with a small contribution from area-level socioeconomic disadvantage. These findings suggests that mental health planning and intervention activities will likely deliver greatest benefits by focusing on individual-level determinants, correlates, and mediators of disease burden and inequality associated with psychosocial distress and other mental health outcomes.

## Figures and Tables

**Table 1:** Sample characteristics and prevalence estimates for high psychosocial distress.

Variable	Characteristics		Prevalence	
	N	%	n	%
POSTAL AREA LEVEL				
*Walkability*				
Low	25,217	27.7	1983	7.9
Low-medium	31,023	34.0	2440	7.9
Medium-high	19,232	21.1	1548	8.0
High	15,670	17.2	1154	7.4
*Socioeconomic disadvantage*				
Q1—Most	17,153	18.8	2096	12.2
Q2	19,272	21.1	1800	9.3
Q3—Middling	14,833	16.3	1109	7.5
Q4	19,789	21.7	1177	5.9
Q5—Least	20,095	22.0	943	4.7
INDIVIDUAL LEVEL				
*Sex*				
Male	44,220	48.5	3008	6.8
Female	46,922	51.5	4117	8.8
*Age*				
45–49	13,480	14.8	1328	9.9
50–54	16,619	18.2	1587	9.5
55–59	16,601	18.2	1367	8.2
60–64	13,611	14.9	938	6.9
65–69	10,093	11.1	536	5.3
70–74	6792	7.5	361	5.3
75–79	4898	5.4	319	6.5
80–84	6432	7.1	435	6.8
85+	2616	2.9	254	9.7
*Language spoken at home*				
English	77,307	84.8	5230	6.8
Other	13,835	15.2	1895	13.7
*Education level*				
Less than secondary school	7236	7.9	1176	16.3
Secondary school graduation	26,355	28.9	2267	8.6
Trade, certificate or diploma	28,678	31.5	2044	7.1
University degree	28,873	31.7	1638	5.7
*Relationship status*				
Partner	68,138	74.8	4457	6.5
No partner	23,004	25.2	2668	11.6
*Employment status*				
Full-time work	32,578	35.7	2052	6.3
Part-time work	13,122	14.4	996	7.6
Other work	1319	1.4	168	12.7
Not working	44,123	48.4	3909	8.9
*Health insurance type*				
Private with extras	53,835	59.1	3054	5.7
Private without extras	12,822	14.1	746	5.8
Government health care card	11,656	12.8	1974	16.9
None	12,829	14.1	1351	10.5
*Smoking status*				
Never smoked	53,560	58.8	3662	6.8
Past smoker	31,276	34.3	2366	7.6
Current smoker	6306	6.9	1097	17.4
*Body mass category*				
Underweight	1247	1.4	177	14.2
Normal weight	35,709	39.2	2467	6.9
Overweight	35,555	39.0	2458	6.9
Obese	18,631	20.4	2023	10.9
*Total physical activity*				
0 min	5296	5.8	912	17.2
1–149 min	15,102	16.6	1635	10.8
150–299 min	15,675	17.2	1185	7.6
≥300 min	55,069	60.4	3393	6.2
*Diagnosed chronic conditions*				
0	31,050	34.1	1397	4.5
1	36,544	40.1	2487	6.8
2	17,915	19.7	2049	11.4
3 or more	5633	6.2	1192	21.2
*Treated chronic conditions*				
0	41,261	45.3	2683	6.5
1	29,791	32.7	2217	7.4
2	14,285	15.7	1363	9.5
3 or more	5805	6.4	862	14.8
*Limited physical functioning*				
None	32,198	35.3	1353	4.2
Minor	24,974	27.4	1169	4.7
Moderate	20,074	22.0	1798	9.0
Severe	13,896	15.2	2805	20.2

**N** Stratum total, **n** Stratum outcome frequency, **%** Stratum outcome per cent.

**Table 2:** Conditional auto regression model summaries for high psychosocial distress.

Individual-Level Adjustment	Model 1	Model 2	Model 3	Model 4	Model 5
	No	Yes	Yes	Yes	Yes
*Prevalence ratios (95% CrI)*					
Constant	0.99 (0.96–1.02)	0.99 (0.97–1.02)	0.97 (0.91–1.03)	1.07 (1.02–1.12)	1.04 (0.97–1.12)
Walkability					
Low	–	–	1.00	–	1.00
Low-medium	–	–	1.01 (0.94–1.08)	–	1.00 (0.94–1.07)
Medium-high	–	–	1.08 (0.99–1.18)	–	1.07 (0.99–1.16)
High	–	–	1.03 (0.93–1.15)	–	1.03 (0.94–1.13)
Socioeconomic disadvantage					
Q1—Most	–	–	–	1.00	1.00
Q2	–	–	–	0.98 (0.91–1.04)	0.98 (0.91–1.05)
Q3— Middling	–	–	–	0.92 (0.86–1.00)	0.92 (0.85–0.99)
Q4	–	–	–	0.90 (0.83–0.98)	0.90 (0.83–0.97)
Q5—Least	–	–	–	0.82 (0.76–0.90)	0.83 (0.76–0.90)
*Model diagnostics*					
pD	127.85	21.73	24.40	15.32	17.20
DIC	1557.25	1418.33	1419.26	1409.06	1410.40
Spatial fraction	0.99	0.88	0.88	0.61	0.55

**CrI** credible interval, **pD** effective parameters, **DIC** Deviance Information Criterion. **Model 1** null model with expected cases proportional to the overall prevalence. **Model 2** null model with expected cases adjusted for individual-level factors. **Model 3** Model 2 + Sydney Walkability Index. **Model 4** Model 2 + Index of Relative Socioeconomic Disadvantage. **Model 5** Model 3 + Index of Relative Socioeconomic Disadvantage.

**Table 3:** Unadjusted and fully-adjusted odds ratios for individual-level adjustment variables.

	Unadjusted		Adjusted	
	OR	95% CI	OR	95% CI
*Sex*	*p* < 0.0001		*p* = 0.2434	
Male	1.00		1.00	
Female	1.32	1.25–1.38	0.97	0.91–1.02
*Age*	*p* < 0.0001		*p* < 0.0001	
45–49	1.00		1.00	
50–54	0.97	0.89–1.04	0.82	0.76–0.89
55–59	0.82	0.76–0.89	0.57	0.52–0.62
60–64	0.68	0.62–0.74	0.36	0.32–0.39
65–69	0.51	0.46–0.57	0.21	0.18–0.24
70–74	0.51	0.46–0.58	0.16	0.14–0.18
75–79	0.64	0.56–0.72	0.16	0.14–0.19
80–84	0.66	0.59–0.74	0.13	0.12–0.15
85+	0.98	0.85–1.13	0.14	0.12–0.17
*Language spoken at home*	*p* < 0.0001		*p* < 0.0001	
English	1.00		1.00	
Other	2.19	2.07–2.31	1.92	1.80–2.04
*Education level*	*p* < 0.0001		*p* < 0.0001	
Less than secondary school	3.23	2.98–3.50	1.70	1.55–1.87
Secondary school graduation	1.56	1.47–1.67	1.20	1.12–1.29
Trade, certificate or diploma	1.28	1.19–1.36	1.09	1.02–1.18
University degree	1.00		1.00	
*Relationship status*	*p* < 0.0001		*p* < 0.0001	
Partner	1.00		1.00	
No partner	1.87	1.78–1.97	1.41	1.33–1.50
*Employment status*	*p* < 0.0001		*p* < 0.0001	
Full-time work	1.00		1.00	
Part-time work	1.22	1.13–1.32	1.14	1.05–1.24
Other work	2.17	1.84–2.57	1.57	1.30–1.89
Not working	1.45	1.37–1.53	1.46	1.35–1.58
*Health insurance type*	*p* < 0.0001		*p* < 0.0001	
Private with extras	1.00		1.00	
Private without extras	1.03	0.95–1.12	1.03	0.94–1.12
Government health care card	3.39	3.19–3.60	1.78	1.65–1.92
None	1.96	1.83–2.09	1.36	1.27–1.47
*Smoking status*	*p* < 0.0001		*p* < 0.0001	
Never smoked	1.00		1.00	
Past smoker	1.12	1.06–1.18	1.07	1.00–1.13
Current smoker	2.87	2.67–3.09	1.64	1.51–1.78
*Body mass category*	*p* < 0.0001		*p* < 0.0001	
Underweight	2.23	1.89–2.63	1.61	1.34–1.93
Normal weight	1.00		1.00	
Overweight	1.00	0.94–1.06	0.93	0.87–0.99
Obese	1.64	1.54–1.75	0.88	0.82–0.94
*Total physical activity*	*p* < 0.0001		*p* < 0.0001	
0 min	1.00		1.00	
1–149 min	0.58	0.53–0.64	0.75	0.68–0.82
150–299 min	0.39	0.36–0.43	0.64	0.58–0.71
≥300 min	0.32	0.29–0.34	0.58	0.53–0.64
*Diagnosed chronic conditions*	*p* < 0.0001		*p* < 0.0001	
0	1.00		1.00	
1	1.55	1.45–1.66	1.56	1.45–1.68
2	2.74	2.55–2.94	2.45	2.26–2.66
3 or more	5.70	5.24–6.19	4.32	3.90–4.78
*Treated chronic conditions*	*p* < 0.0001		*p* < 0.0240	
0	1.00		1.00	
1	1.16	1.09–1.23	1.02	0.96–1.10
2	1.52	1.42–1.62	1.01	0.93–1.10
3 or more	2.51	2.31–2.72	1.17	1.05–1.29
*Limited physical functioning*	*p* < 0.0001		*p* < 0.0001	
None	1.00		1.00	
Minor	1.12	1.03–1.21	1.24	1.15–1.35
Moderate	2.24	2.09–2.41	2.15	1.98–2.33
Severe	5.77	5.38–6.17	4.41	4.05–4.79

**OR** Odds ratio, **CI** Confidence interval

**Table 4:** Conditional auto regression model summaries for sensitivity analyses.

	Baseline	Walkability
*Prevalence ratios (95% CrI)*		
Constant	0.97 (0.97–1.02)	0.97 (0.91–1.03)
Walkability		
Low	–	1.00
Low-medium	–	1.01 (0.94–1.08)
Medium-high	–	1.08 (0.99–1.18)
High	–	1.03 (0.93–1.15)
*Model diagnostics*		
pD	23.58	25.97
DIC	1420.05	1420.99
Spatial fraction	0.90	0.90

**CrI** credible interval, **pD** effective parameters, **DIC** Deviance Information Criterion. **Baseline** null model with adjusted offsets EXCLUDING individual physical activity level. **Walkability** Baseline + Sydney Walkability Index.
